# Disruption of Cell Adhesion and Cytoskeletal Networks by Thiol-Functionalized Silica-Coated Iron Oxide Nanoparticles

**DOI:** 10.3390/ijms21249350

**Published:** 2020-12-08

**Authors:** Karel Královec, Lucie Melounková, Marcela Slováková, Nikola Mannová, Miloš Sedlák, Jan Bartáček, Radim Havelek

**Affiliations:** 1 Department of Medical Biochemistry, Faculty of Medicine in Hradec Králové, Charles University, Šimkova 870, 500 03 Hradec Králové, Czech Republic; Karel.Kralovec@upce.cz (K.K.); melounkl@lfhk.cuni.cz (L.M.); 2 Department of Biological and Biochemical Sciences, Faculty of Chemical Technology, University of Pardubice, Studentská 573, 532 10 Pardubice, Czech Republic; Marcela.Slovakova@upce.cz (M.S.); nikola.mannova@student.upce.cz (N.M.); 3 Institute of Organic Chemistry and Technology, Faculty of Chemical Technology, University of Pardubice, Studentská 573, 532 10 Pardubice, Czech Republic; milos.sedlak@upce.cz (M.S.); jan.bartacek@upce.cz (J.B.)

**Keywords:** magnetic nanoparticles, cytotoxicity, cytoskeleton, cell adhesion, focal adhesion kinase

## Abstract

One of the major obstacles that limits the use of magnetic nanoparticles in biomedical applications is their potential toxicity. In the present study, we evaluated the cytotoxic effects of thiol-functionalized silica-coated iron oxide (Fe_3_O_4_@SiO_2_-SH) nanoparticles using human lung epithelial cells A549. We investigated the effect of Fe_3_O_4_@SiO_2_-SH nanoparticles on the cell viability, proliferation, cell cycle distribution, adhesion, apoptosis, and the orientation of the cytoskeletal networks, as well as on expression of proteins involved in cell death, cell survival, and cell adhesion. We demonstrated that exposure of A549 cells to Fe_3_O_4_@SiO_2_-SH nanoparticles resulted in severe disruption of the actin microfilaments and microtubule cytoskeleton and reduced the size of focal adhesions. Furthermore, cell adhesion was significantly affected as well as the phosphorylation of focal adhesion kinase (FAK), extracellular-signal-regulated kinase (ERK), and p38. Our findings highlight the need for in-depth cytotoxic evaluation of nanoparticles supporting their safer use, especially in biomedical applications.

## 1. Introduction

Over the past decades, magnetic nanoparticles (MNPs) have been widely studied with respect to possible use in medical and biomedical applications, especially as contrast agents and tracers for cell labelling and cell tracking [1,2] by magnetic resonance imaging (MRI) [3,4] and magnetic particle imaging (MPI) [5], as magnetic carriers for targeted drug delivery [6,7] and cell separation [8,9], and as heating agents for magnetic fluid hyperthermia [10,11]. These applications either assume efficient uptake of MNPs by cells or outcomes of these applications can be significantly enhanced by the internalization of particles into cells. Numerous studies have thus examined various types of coatings not only to achieve high colloidal stability under physiological conditions and biocompatibility but also to pave a way for MNPs into cells, i.e., to provide a platform for the optimization of their cellular uptake [12]. Among the plethora of procedures suggested, the encapsulation of magnetic nanoparticles into silica and its organically modified counterparts shows great potential due to the high colloidal stability of silica-coated particles in water, chemical stability, and biological inertness of silica, ease of encapsulation and ease of further covalent functionalization, including attachment of antibodies, nucleic acids, and other types of advanced biofunctionalization [13,14,15].

Silica can be covalently modified almost with any chemical moiety by utilizing a suitable organoalkoxysilane, which integrates the desired moiety into a growing silica shell or that attaches the moiety to the silica surface (see, e.g., [16,17]). Organic functionalization with simple organoalkoxysilanes, most typically organotrialkoxysilanes, can introduce various chemical groups on the surface of silica or silica-coated nanoparticles, modifying their surface chemistry, altering zeta potential, introducing new chemical equilibria, or just providing functional groups for coupling reactions. Similarly, more complex organosilanes can attach fluorescent tags, specific binding sites, or other functionalities. Importantly, the described functionalization procedures, based on the hydrolysis and condensation reactions of organoalkoxysilanes, can be controlled very well, i.e., well-defined and homogeneous nanoparticles with core-shell morphology and controlled density of functional groups can be achieved.

The thiol group, providing acidobasic and redox reactions and offering an excellent platform for further functionalization [18,19,20], is an interesting choice that has become popular in surface modification of nanoparticles but whose effects on cells, however, are not fully understood. Several studies have shown that iron oxide MNPs coated with thiol-containing molecules, such as mercaptosuccinic acid (MSA) or dimercaptosuccinic acid (DMSA), increase biocompatibility [21,22,23] and intracellular uptake [24,25]. Moreover, DMSA-coated iron oxide nanoparticles with doxorubicin have shown a strong cytotoxic effect against breast cancer (MDA-MB-231) cells, which means that thiolated nanoparticles can be used for efficient intracellular delivery of specific biologically active molecules [26]. Nevertheless, such an approach relies on the internalization of a larger amount of thiolated nanoparticles into cells, and, inevitably, a proven concern arises over the impacts of high intracellular concentrations of these nanoparticles on cellular functions. Although there are numerous reports concerning the cytotoxicity of thiolated particles [21,22,23,24,25], there is very little information on their effects on the cytoskeletal networks and other members of the cell adhesion complex [27]. Moreover, no studies to date have specifically focused on the cytotoxicity of thiol-functionalized silica-coated iron oxide (Fe_3_O_4_@SiO_2_-SH) nanoparticles, which represent a well-defined and reproducible model system with thiol groups.

The present study is devoted to the in vitro interactions between Fe_3_O_4_@SiO_2_-SH nanoparticles and A549 cells; specifically, it aims to investigate the effects of thiolated nanoparticles on the cytoskeletal networks and cell adhesion, cell viability, proliferation, cell cycle distribution, apoptosis, and expression of proteins involved in cell death, cell survival, and cell adhesion. The understanding of nanoparticles-cell interactions will contribute to the development of safe and efficient nanoparticle agents for biomedical applications, which is of paramount importance.

## 2. Results

### 2.1. Preparation and Characterization of Fe_3_O_4_@SiO_2_-SH Nanoparticles

**Figure 1:**
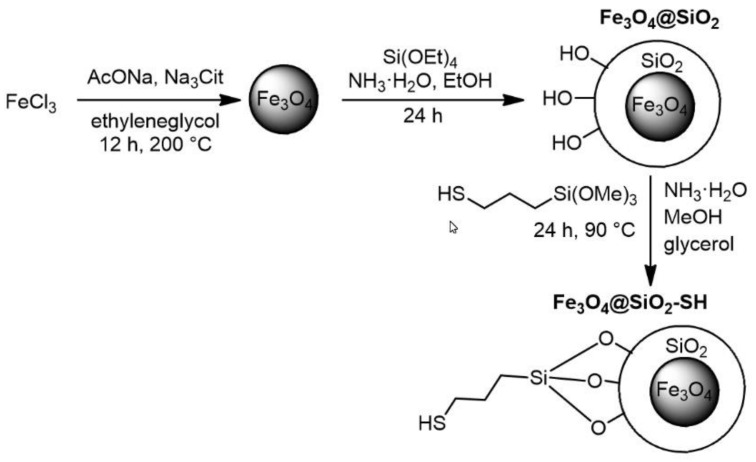
Three-step procedure for preparation of thiol-functionalized silica-coated iron oxide (Fe_3_O_4_@SiO_2_-SH) nanoparticles.

The Fe_3_O_4_@SiO_2_-SH nanoparticles were prepared by the three-step procedure schematically illustrated in Figure 1. At first, magnetite nanoparticles were obtained by solvothermal reduction of FeCl_3_ in the presence of ethylene glycol according to published procedure [28]. After purification, the nanoparticles were coated with silica layer by modified Stöber process [28,29]. The final functionalization of so obtained Fe_3_O_4_@SiO_2_ particles was carried out by reaction with (3-mercaptopropyl) trimethoxysilane according to [30], whose hydrolysis and subsequent condensation with silanol groups at the surface of Fe_3_O_4_@SiO_2_ particles led to the covalent modification of the silica layer.

**Figure 2:**
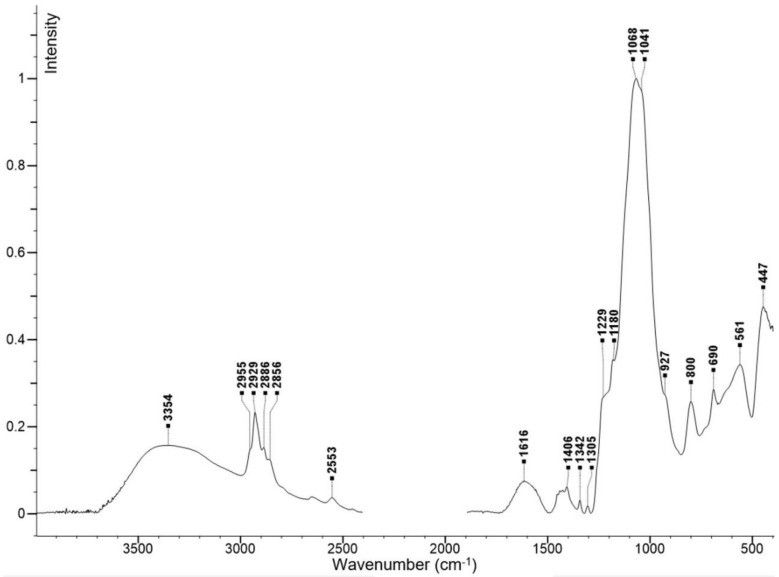
FT-IR spectra of prepared Fe_3_O_4_@SiO_2_-SH nanoparticles.

The automated elemental analysis of the final product showed sulfur content of 11% by weight. Fourier transform infrared spectroscopy (FT-IR) analysis (see the spectrum in Figure 2) was in agreement with the expected structure and reference data [30]. Specifically, the broad band with maximum at ≈3354 cm^−1^ corresponded to the O–H stretching vibration from silanol groups. In the range of 2955–2856 cm^−1^ C–H stretching vibrations within CH_2_ groups of the 3-mercaptopropyl fragment were propagated while the S–H stretching vibration was manifested at 2553 cm^−1^. The strongest band at 1068 cm^−1^ corresponds to the asymmetric stretching vibration of Si–O–Si arrangement. (Figure 2).

**Figure 3:**
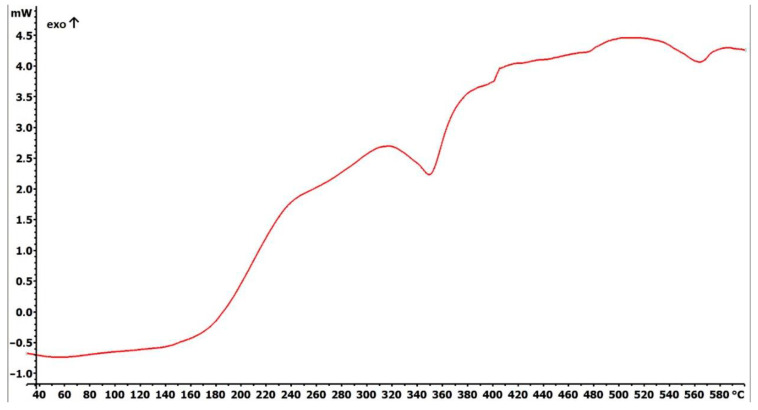
Differential scanning calorimetry (DSC) thermogram of prepared Fe_3_O_4_@SiO_2_-SH nanoparticles.

Differential scanning calorimetry (DSC) analysis showed thermal stability up to 140 °C, followed by gradual exothermic effects, which might be attributed to decomposition of mercaptopropyl moieties (Figure 3).

**Figure 4:**
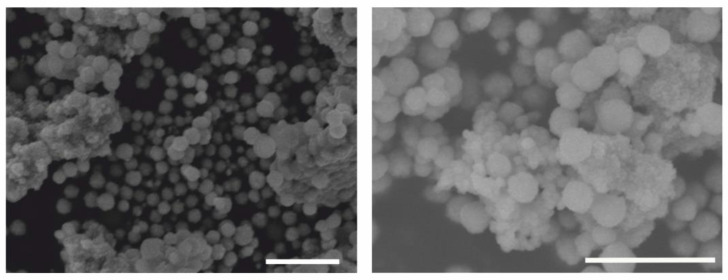
Representative SEM images of Fe_3_O_4_@SiO_2_-SH nanoparticles. The scale bars are 1 µm in both SEM micrographs.

The morphology and size of the Fe_3_O_4_@SiO_2_-SH nanoparticles were studied by scanning electron microscopy (SEM). The images at different magnifications showed that the prepared nanoparticles have a spherical character. During the drying of the nanoparticle suspension prior to the SEM analysis, only a minor fraction of aggregates with a porous structure was formed (Figure 4). Individual particles within the sample were characterized by a mean size of 193 ± 34 nm.

The hydrodynamic diameter of the Fe_3_O_4_@SiO_2_-SH nanoparticles was determined by means of dynamic light scattering (DLS) in both deionized water and complete cell culture medium in order to investigate the colloidal behavior in relevant experimental conditions. The Z average hydrodynamic size in deionized water was 458 ± 9 nm and polydispersity index (PI) 0.410 ± 0.031. The Z average hydrodynamic size in the complete cell culture medium was 559 ±13 nm and PI 0.34 ± 0.04. Finally, the measurement of the zeta potential provided −68 ± 4 mV for deionized water and −84 ±17 mV for complete cell culture medium.

### 2.2. Effects of Fe_3_O_4_@SiO_2_-SH Nanoparticles on Proliferation and Viability

**Figure 5:**
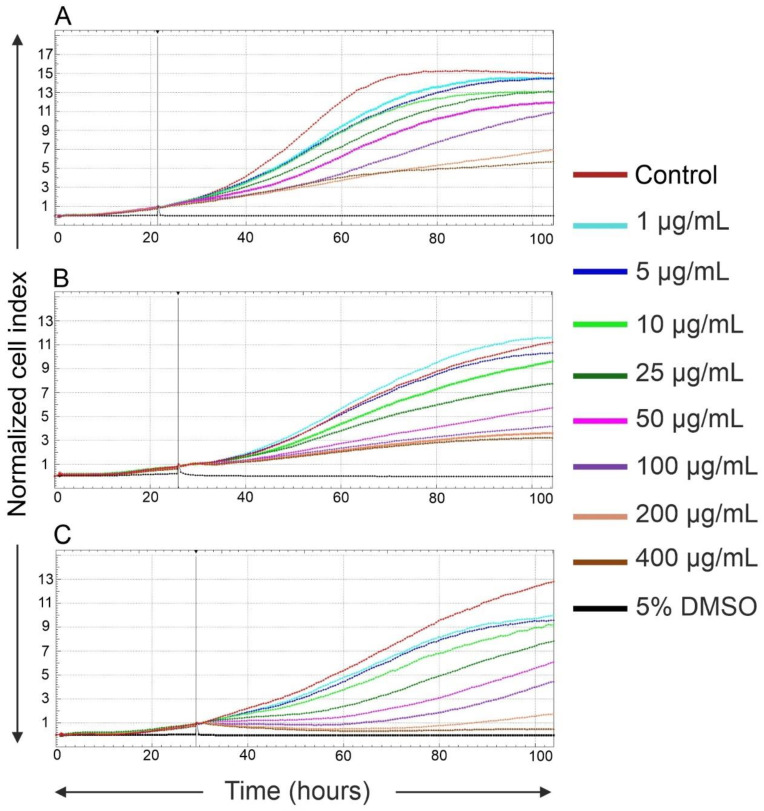
Growth kinetics of human A549 (**A**), HepG2 (**B**), and MCF-7 (**C**) cells treated with Fe_3_O_4_@SiO_2_-SH nanoparticles at 1, 5, 10, 25, 50, 100, 200, and 400 µg/mL—dynamic real-time monitoring of proliferation and cytotoxicity by the xCELLigence system dedicated to adherent cell lines. The black vertical line indicates the time point when the tested nanoparticles were added. Cells treated with 5% H_2_O were used as vehicle control, whereas cells treated with 5% dimethyl sulfoxide (DMSO) were used as a positive control. The normalized cell index was measured over 72 h. Plots shown are representative of at least three replicate experiments in each case.

The cell viability and proliferation A549 cells incubated with Fe_3_O_4_@SiO_2_-SH nanoparticles were assessed in real time by using the label-free xCELLigence system. The xCELLigence system allows continuous monitoring of cell adhesion, morphology, viability, and rate of cell proliferation based on measurement of impedance, which is displayed as normalized cell index (CI) values. As shown in Figure 5, the application of Fe_3_O_4_@SiO_2_-SH nanoparticles decreased the proliferation rate of A549, HepG2, and MCF-7 cells in dose-dependent manners within 72 h of treatment; the decrease was more pronounced in the case of MCF-7 cells. Exposure to Fe_3_O_4_@SiO_2_-SH nanoparticles at 200 μg/mL and 400 μg/mL resulted in the almost complete inhibition of cell proliferation with a permanently decreased cell index.

**Figure 6:**
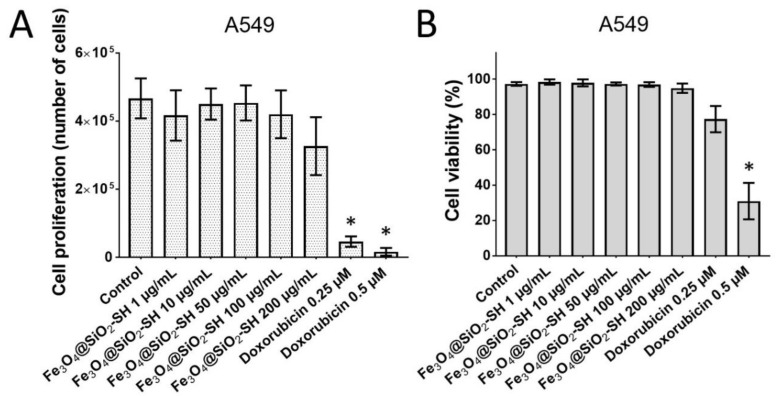
The effect of Fe_3_O_4_@SiO_2_-SH nanoparticles on the proliferation (**A**) and viability (**B**) of human A549 lung carcinoma cells. The changes in the proliferation and viability were determined 48 h after the treatment by using Trypan blue exclusion analysis. Results are shown as the mean ± SD from three experiments. *—significantly different from the control (*p* ≤ 0.05). Cells treated with 0.25 and 0.5 µM doxorubicin were used as a positive control.

To further explore whether the treatment with Fe_3_O_4_@SiO_2_-SH nanoparticles truly inhibits the cell growth and survival of human cells; we examined cytostatic and cytotoxic effects of these nanoparticles at different concentrations (1, 10, 50, 100, and 200 µg/mL) on A549 cells by Trypan blue exclusion analysis. According to the results in Figure 6A, 48 h of exposure at given concentrations did not significantly reduce proliferation of Fe_3_O_4_@SiO_2_-SH-treated cells compared to sham-treated negative control cells. Meanwhile, the reference antineoplastic drug doxorubicin applied at 0.25 and 0.5 µM concentrations was found more cytostatic (*p* ≤ 0.05) in parallel treatments. Moreover, we examined the effect of Fe_3_O_4_@SiO_2_-SH nanoparticles on A549 cells viability at 48-h intervals of treatment. As shown in Figure 6B, the viability of A549 cells was not considerably affected by the treatment with the nanoparticles within 1–200 µg/mL dose range. On the other hand, doxorubicin at 0.5 µM had clear negative impact on A549 cells survival and significantly decreased percentage of viable cells (*p* ≤ 0.05).

### 2.3. Effects of Fe_3_O_4_@SiO_2_-SH Nanoparticles on the Cell Cycle Progression of A549 Cells

**Figure 7:**
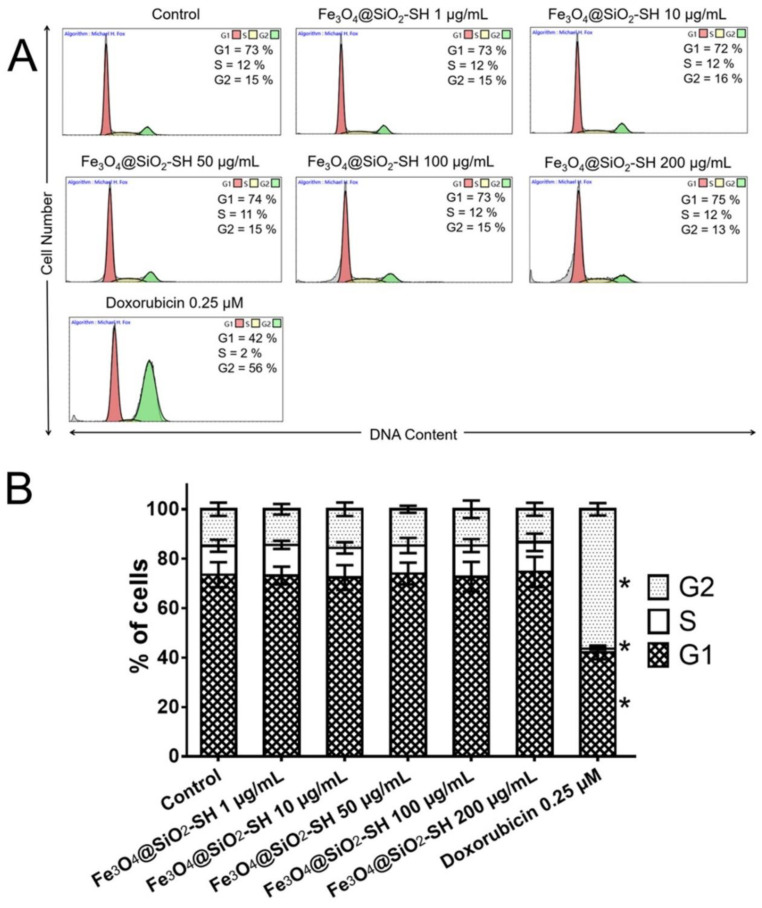
Analysis of the cell cycle after the treatment with Fe_3_O_4_@SiO_2_-SH nanoparticles at 1, 10, 50, 100, and 200 µg/mL. (**A**) The figure shows representative histograms of human A549 lung carcinoma cells after the 48 h interval with the mean percentage of cells cycling through phases G1, S, and G2 based on flow cytometry measurement of three separate treatments. (**B**) The bar graph summarizes cumulative data on the percentage of cells in each phase of the cell cycle. Cells treated with 0.25 and 0.5 µM doxorubicin were used as a positive control. Data are presented as the mean values ± SD from three experiments. *—significantly different from the control (*p* ≤ 0.05).

To deepen cell proliferation assessment, the impact of Fe_3_O_4_@SiO_2_-SH nanoparticles on the cell cycle was studied, namely the cell cycle distribution of A549 cells was analyzed. Doxorubicin (positive control) dosed at 0.25 µM for 48 h induced a marked cell cycle blocking with a significant increase of G2/M phase cells. As indicated in Figure 7A, the G1, S, and G2 phases of cells treated with 0.25 µM doxorubicin constituted 42%, 2%, and 56%, respectively, while percentages of negative control were 73% for G1, 12% for S, and 15% for G2. The flow cytometry histograms showing A549-cells distribution at different cell cycle phases and the bar graphs for cumulative data (Figure 7B) clearly revealed that 48-h exposures to Fe_3_O_4_@SiO_2_-SH nanoparticles in dose range of roughly 1–200 µg/mL did not have a significant effect on the cell cycle compared to negative control cells. Consistent with demonstrated apparent lack of antiproliferative activity and no inhibitory effect on cell survival, these data further confirmed that Fe_3_O_4_@SiO_2_-SH nanoparticles at concentrations 1–200 µg/mL had no apparent effects on growth of A549 cells.

### 2.4. Effect of Fe_3_O_4_@SiO_2_-SH Nanoparticles on Cell Cytoskeleton and Focal Adhesions (FAs)

To further elucidate the discrepancy between the Trypan blue and xCELLigence results, we performed immunofluorescence staining of cytoskeletal protein F-actin, β-tubulin, and focal adhesion (FA)-associated protein paxillin. This discrepancy could be explained by alterations of cell morphology due to cytoskeletal changes and/or cell death by apoptosis, which are not accompanied by loss of cellular membrane permeability.

**Figure 8:**
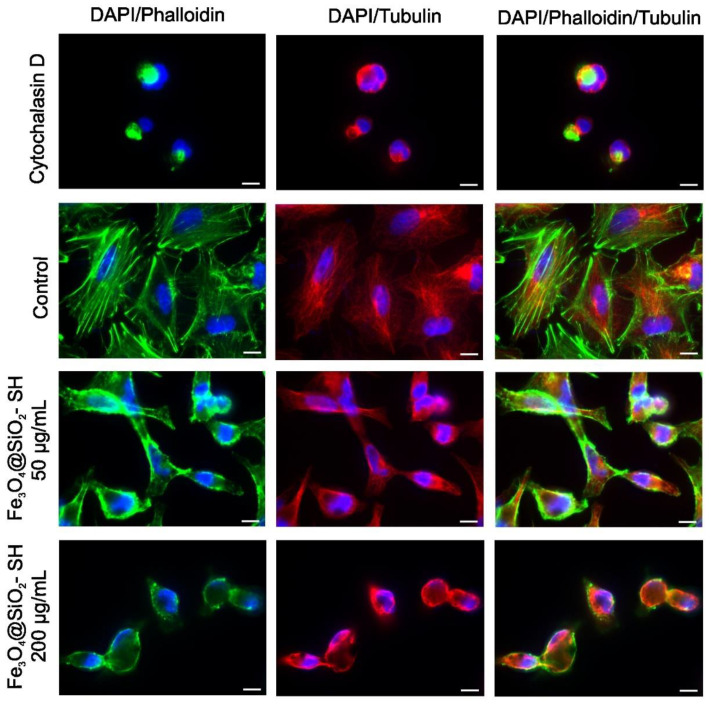
Fluorescent microscopic images of A549 cells stained with Alexa Fluor 488 phalloidin (F-actin, green) and anti-β-tubulin antibody (red), and counterstained with 4′,6-diamidino-2-phenylindole ((DAPI), nuclei, blue). The cells were sham-treated with H_2_O (negative control) or treated Fe_3_O_4_@SiO_2_-SH nanoparticles at 50 and 200 µg/mL. Cells treated with 0.5 µg/mL of cytochalasin D, a member of the cytochalasin fungal alkaloids that acts as a potent inhibitor of actin polymerization, were used as a reference in this assay. Experiments were performed in triplicates, but only photographs from representative chambers are shown. The scale bar in the images represents 10 μm.

**Figure 9:**
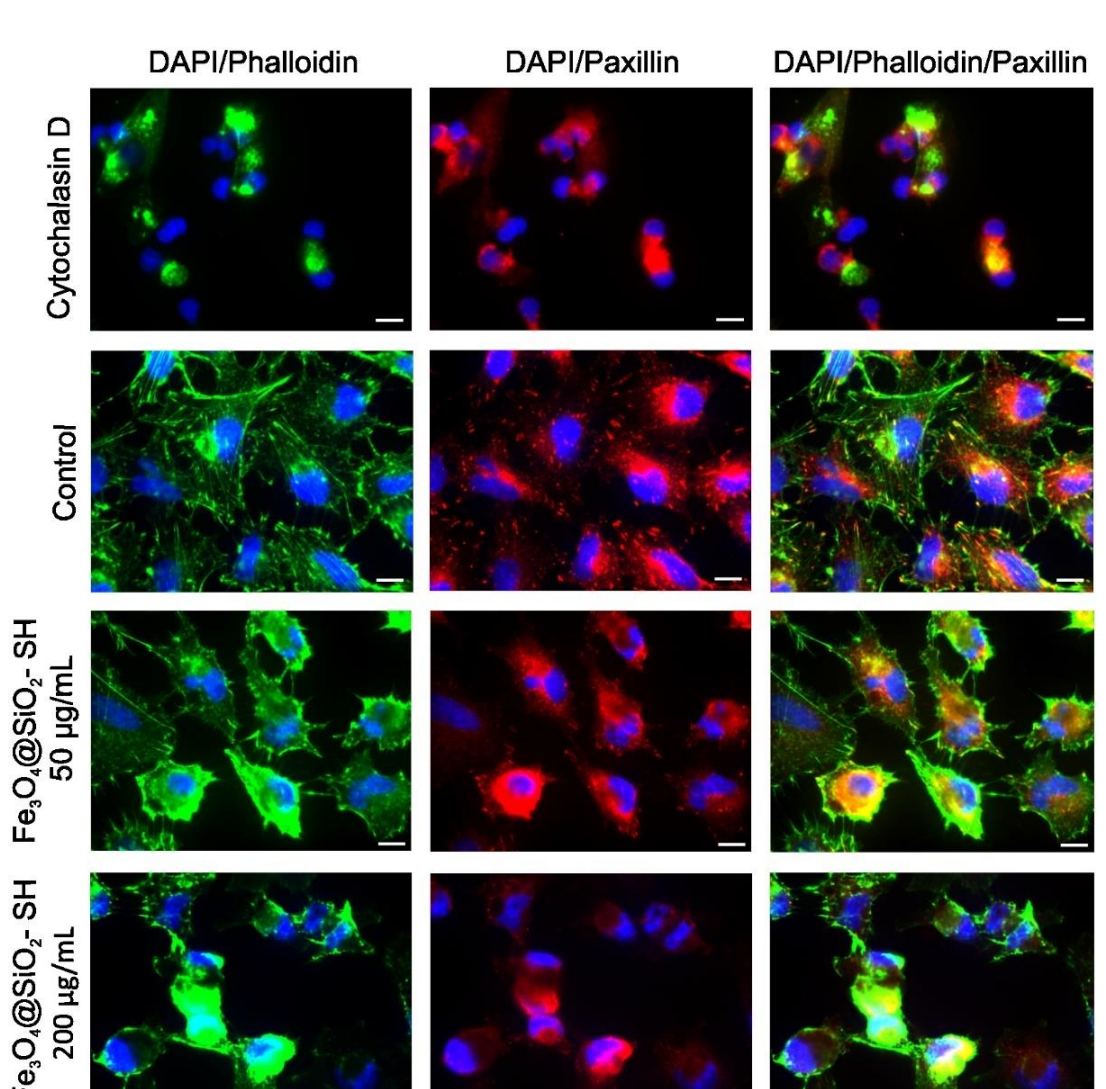
Fluorescent microscopic images of A549 cells stained with Alexa Fluor 488 phalloidin (F-actin, green) and anti-paxillin (red), and counterstained with DAPI (nuclei, blue). The cells were sham-treated with H_2_O (negative control) or treated Fe_3_O_4_@SiO_2_-SH nanoparticles at 50 and 200 µg/mL. Cells treated with 0.5 µg/mL of cytochalasin D, a member of the cytochalasin fungal alkaloids that acts as a potent inhibitor of actin polymerization, were used as a reference in this assay. Experiments were performed in triplicates by using epifluorescence microscopy. Photographs from representative chambers are shown. The scale bar in the images represents 10 μm.

**Figure 10:**
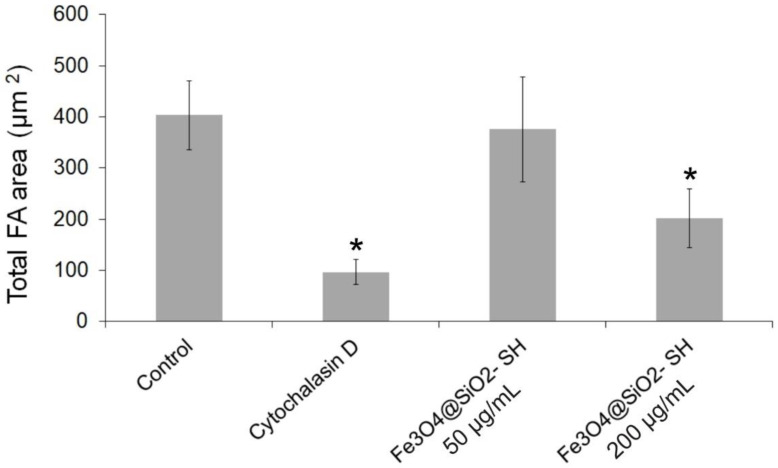
Histogram representing the cellular focal adhesion (FA) areas of control cells or cells treated with Fe_3_O_4_@SiO_2_-SH nanoparticles at 50 and 200 µg/mL. Values are represented as the mean ± SD (*n* = 10). *—significantly different to control (*p* ≤ 0.05). Cells treated with cytochalasin D (0.5 μg/mL) were used as a positive control.

Epifluorescence imaging revealed that mock-treated A549 cells had an extensive actin and microtubule networks (Figure 8). After 24 h incubation with 50 µg/mL and 200 µg/mL of Fe_3_O_4_@SiO_2_-SH nanoparticles, cells exhibited small and round cell morphology, with collapsed microtubule network around the cell nuclei, disrupted orientation of the F-actin network, and a loss of actin stress fibers (Figure 8 and Figure 9). A549 adhesion was assessed by immunofluorescent staining of FA contacts targeting paxillin as a marker for FAs (Figure 9). The results of a follow-up quantitative image analysis confirmed that the total area occupied by FAs ions was also affected when A549 cells were incubated with Fe_3_O_4_@SiO_2_-SH nanoparticles at 200 µg/mL (Figure 10). The smaller size of FAs is indicative of a reduced capability of adhesion to the extracellular matrix (ECM).

### 2.5. Detection of Apoptosis

**Figure 11:**
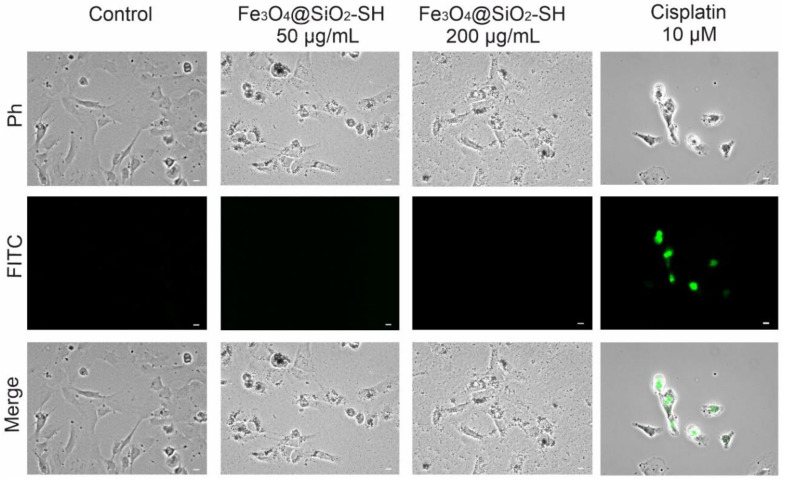
Microscopic study of the activation of caspase-3/7 in A549 cells. The cells were exposed to Fe_3_O_4_@SiO_2_-SH nanoparticles at 50 and 200 µg/mL for 24 h. The images showed apoptotic signals of a CellEvent™ Caspase-3/7 Green Detection Reagent (apoptotic cells, green, FITC), corresponding optical phase-contrast microscopy (Ph), and merged images (Merge). Cells sham-treated with H_2_O (negative control) were used as a negative control, whereas cells treated with 10 µM cisplatin represented the positive control. Photographs are representative pictures from three independent experiments. The scale bar in the images represents 10 μm.

To explore the possible mechanism of cytotoxicity of Fe_3_O_4_@SiO_2_-SH nanoparticles, we investigated the activation of caspase-3/7 as a hallmark of the cell death mediated by apoptosis. As shown in Figure 11, this characteristic marker of apoptosis was not found after the treatment of A549 cells with 50 μg/mL and 200 μg/mL Fe_3_O_4_@SiO_2_-SH nanoparticles for 24 h.

### 2.6. Effects of Fe_3_O_4_@SiO_2_-SH Nanoparticles on the Selected Proteins Involved in Cell Cycle Progression and FA Signaling

**Figure 12:**
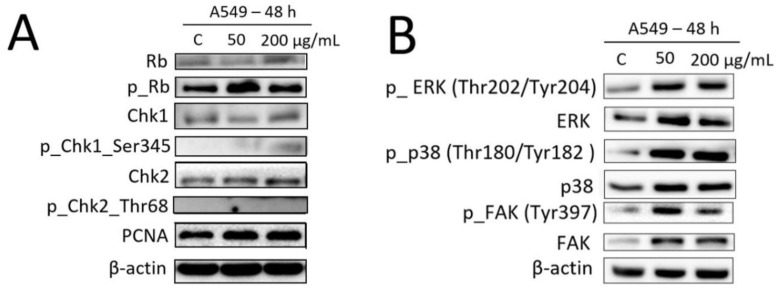
Western blot analysis of proteins that regulate cell cycle progression (**A**) and cell adhesion (**B**) in A549 lung carcinoma cells upon treatment with Fe_3_O_4_@SiO_2_-SH nanoparticles at 50 and 200 µg/mL for 48 h. Negative control cells were mock treated with 5% phosphate-buffered saline (PBS), indicated as C. These experiments were performed at least three times with similar results, and a cropped blot is shown from one representative experiment.

To verify the results of cell cycle analysis, we first determined the level of proteins involved in cell cycle control in A549 cells treated with 50 and 200 μg/mL of Fe_3_O_4_@SiO_2_-SH nanoparticles 48 h. As shown in Figure 12A, there was no upregulation or downregulation in proteins that promote the growth arrest via ataxia telangiectasia and Rad3-related protein kinase/checkpoint kinase 1 (ATR/Chk1), ataxia-telangiectasia mutated kinase/checkpoint kinase 2 (ATM/Chk2), or retinoblastoma protein (Rb) signalling axis. These data, together with the results of cell counts made by Trypan blue exclusion staining and cell cycle analysis made by flow cytometry, showed that Fe_3_O_4_@SiO_2_-SH nanoparticles exerted no inhibitory effects on the proliferation of A549 cells.

Since the A549 cells treated Fe_3_O_4_@SiO_2_-SH nanoparticles underwent no noticeable change in growth, the microscopic examinations revealed morphological changes associated with marked impairment in the spatial organization of microfilaments, microtubules, and the FA-associated protein paxillin. To explore the underlying mechanisms involved in these morphological changes, the extracellular-signal-regulated kinase (ERK), p38 MAP kinase (p38), and focal adhesion kinase (FAK) protein expression levels and its activation were determined by Western blotting. As illustrated in Figure 12B, treatments with Fe_3_O_4_@SiO_2_-SH nanoparticles for 48 h caused considerable increase in the FAK protein level, while the amounts of ERK and p38 were less robustly increased under the same treatment conditions. On the other hand, the exposure to Fe_3_O_4_@SiO_2_-SH nanoparticles distinctly induced the phosphorylation of FAK, as well as ERK and p38.

## 3. Discussion and Conclusions

In the present study, we investigated the effects of Fe_3_O_4_@SiO_2_-SH nanoparticles on the proliferation, viability, adhesion, and the arrangement of cytoskeletal structures, as well as on the selected proteins involved cell cycle progression and FA signaling of A549 cells. Besides the Trypan blue exclusion test for cell viability and proliferation, the inhibitory effect of Fe_3_O_4_@SiO_2_-SH nanoparticles on A549 cells was assessed in real time by using the label-free xCELLigence system. The xCELLigence system allows continuous monitoring of cell adhesion, morphology, viability, and proliferation of cells [31,32]. Our experiments showed that no effects of Fe_3_O_4_@SiO_2_-SH nanoparticles at concentrations ranging from 1 to 200 µg/mL on cell growth could be observed in the A549 cells by both the Trypan blue counting and cell cycle analysis after 48 h. As regards the cell viability under the same experimental conditions, no toxic effects of Fe_3_O_4_@SiO_2_-SH nanoparticles were noted for A549 cells. In Western blot analysis, the lack of growth inhibition in A549 cells by Fe_3_O_4_@SiO_2_-SH nanoparticles after 48 h incubation was demonstrated by unaltered conduction of Chk1/Chk2 signaling pathways, which maintain the genome’s fidelity by blocking the cell cycle after DNA damage [33]. In addition, the exposure to the thiolated nanoparticles had no effect on phosphorylation of pRB at Serine 807 and Serine 811, which is mediated by the kinase activity of cyclin-dependent kinases (Cdks)/cyclin complexes and is essential for proper cell cycle progression [34]. However, as shown in the xCELLigence results, the CI decreased considerably in response to Fe_3_O_4_@SiO_2_-SH nanoparticles, indicating reduced cell adhesion and altered cell morphology.

Fluorescence microscopy observations of F-actin and β-tubulin staining revealed obvious cytoskeleton alterations and changes in A549 cells exposed for 24 h to Fe_3_O_4_@SiO_2_-SH nanoparticles at 200 µg/mL. The disorganized cytoskeletal structures might be the result of spatial hindrance caused by high intracellular nanoparticle concentrations [35] or/and result from direct interaction with cytoskeletal structures through which cells mechanically interact with the extracellular substrate [36]. The actin cytoskeleton plays a crucial role in cell spreading, migration, and adhesion, and is coupled to the ECM via multi-protein complexes called FAs. FAs provide the main sites of cell adhesion to the ECM and associate with the actin cytoskeleton and adaptor/signaling proteins such as paxillin. Paxillin is a key scaffolding protein between the FAs and the actin cytoskeleton [37].

Moreover, the FA dynamics are dependent on microtubules function [38], and the FA can serve as the hot spot for crosstalk between microtubules and actin networks [39]. We therefore examined FAs of A549 cells via immunofluorescent staining of the FA-associated protein paxillin. We observed a marked delocalization of paxillin from FAs and a decrease in the size of FAs after the treatment with Fe_3_O_4_@SiO_2_-SH nanoparticles. This was first observed by using xCELLigence system as decreased CI, which indicated impaired cell adhesion to the ECM as a result of smaller FAs. The decreased CI values, as a result of smaller FAs, were also observed after treatment with titanium dioxide nanoparticles [40] and Ga-substituted ε-Fe_2_O_3_ nanoparticles [41].

To further determine whether the alternations in cellular adhesion may be involved in the activity of Fe_3_O_4_@SiO_2_-SH nanoparticles, selected regulatory proteins were detected by Western blotting. In view of this, 48-h treatment with 50 and 200 µg/mL dose of Fe_3_O_4_@SiO_2_-SH nanoparticles was found to increase FAK, ERK, and p38 phosphorylation. FAK is one of the key components found in FAs of cultured cells, influencing cell adhesion, proliferation, spreading, and migration [42,43,44]. In response to integrin clustering achieved by cell adhesion, the FAK becomes activated via autophosphorylation at Tyr-397, allowing association with Src family member kinases, and synergistically phosphorylates downstream targets such as proteins involved in FA complexes formation (talin, tensin, paxillin) [35,45]. The increased phosphorylation of FAK at Tyr-397 seen in A549 cells might be caused by impaired FAs associated with disrupted actin and microtubule cytoskeleton networks. Consistently, the impaired FAs caused by the disrupted cytoskeletal networks (especially the microtubules) and consequently the increased phosphorylation of FAK was recently reported in Saos-2 cells after titanium dioxide nanoparticles treatment [40]. Additionally, FAK initiates various downstream intracellular signaling pathways in response to adhesion, including mitogen-activated protein kinases (MAPK) [46,47]. Therefore, we detected the expression and activation of two branches in the MAPK signaling pathway, namely ERK1/2 and p38.

Likewise, the phosphorylation of ERK at Thr-202 and Tyr-204 was also significantly increased after 48 h of the treatment of A549 cells with Fe_3_O_4_@SiO_2_-SH nanoparticles. It has been reported that ERK activation affects cytoskeleton organization and provokes focal complexes disassembly during hypoxia/reoxygenation, and this study provides the clue that treating cells with the ERK1/2 inhibitor U0126 improved actin and tubulin cytoskeleton structure, reduced cell contraction, and prevented paxillin redistribution [48]. Therefore, we can suppose that the increased phosphorylation of ERK seen in our experiments is a result of disrupted cytoskeletal networks and reduced FAs size after treatment with Fe_3_O_4_@SiO_2_-SH nanoparticles.

We demonstrated that exposure A549 cells to Fe_3_O_4_@SiO_2_-SH nanoparticles resulted in severe disruption of the actin microfilaments and microtubule cytoskeleton and reduced the size of FAs. Furthermore, cell adhesion was significantly affected as well as protein expression levels. The affected protein expression levels may further impact other cellular functions. The results of this study emphasize the importance of thoroughly understanding nanoparticle-cell interactions with regard to safe use of these particles in medical applications, especially for targeted cancer therapy.

## 4. Materials and Methods

### 4.1. Preparation of Fe_3_O_4_@SiO_2_-SH Nanoparticles

All starting chemicals and solvents were purchased from Sigma (Sigma–Aldrich, St. Louis, MO, USA) or Lach-ner (Lach-ner, Neratovice, Czech Republic) and were used without further purification. The Fe_3_O_4_@SiO_2_-SH nanoparticles were prepared according to the three-step procedure reported in literature [28,30]. At first, a mixture of FeCl_3_·6H2O (4.05 g), sodium acetate (10 g), and sodium citrate (3.75 g) in ethylene glycol (150 mL) was stirred in an Erlenmeyer flask at 30 °C. After one hour, the solution was filtered and transferred into a Teflon-lined autoclave (Berghof DAB-3) and heated to 200 °C for 12 h. After cooling down, the product was washed with deionized water (2 × 100 mL) and ethanol (3 × 100 mL). The particles were subsequently dispersed in deionized water (50 mL) and transferred into an Erlenmeyer flask with ethanol (1 L). The resulting suspension was agitated by using ultrasound (450 W; 15 min) and heated up to 30 °C. Solution of ammonia was added (10 mL; 25%) followed by tetraethoxysilane (10 mL) after 10 min. The reaction mixture was further stirred at 30 °C. After 24 h, the resulting particles of Fe_3_O_4_@SiO_2_ were washed with ethanol (2 × 150 mL) and methanol (3 × 150 mL), and the purified particles were dispersed in methanol (100 mL; 14.7 mg of Fe_3_O_4_@SiO_2_ per mL). The prepared colloidal solution of Fe_3_O_4_@SiO_2_ nanoparticles (42 mL) was mixed with glycerol (96 mL) and degassed by bubbling argon. The mixture was heated up to 90 °C, and solution of ammonia was added (0.95 mL; 25%) followed by addition of (3-mercaptopropyl)trimethoxysilane (0.45 mL) under argon bubbling. The inert gas was introduced into solution for next 1 h. Then, the argon bubbling was stopped, and the reaction mixture was heated to 90 °C for 24 h. During this period, the reaction mixture was 4 times dispersed in an ultrasonic bath (450 W; 5 min). The resulting Fe_3_O_4_@SiO_2_-SH nanoparticles were washed by methanol (2 × 150 mL) and dried in vacuo at room temperature. This procedure provided 990 mg of the final product.

### 4.2. Fundamental Characterizations of Fe_3_O_4_@SiO_2_-SH Nanoparticles 

Elemental analysis of the Fe_3_O_4_@SiO_2_-SH nanoparticles was carried out on an automated Flash 2000 CHNS Analyzer (Thermo Fisher Scientific, Waltham, MA, USA). The FT-IR spectra were recorded on a FT-IR Nicolet iS50 spectrometer by using the ATR technique. The region of the diamond crystal absorption (1900–2400 cm^−1^) was removed from the measured spectrum, and the ATR correction was applied.

Thermal behavior of the sample was studied by differential scanning calorimetry (DSC) by means of a Mettler-Toledo STARe System DSC 2/700 equipped with a FRS 6 ceramic sensor and cooling system HUBERT TC100-MT RC 23. The sample was measured in an open aluminum crucible under nitrogen atmosphere. The DSC curve was recorded with a scanning rate of 3 °C/min within the temperature range of 25–640 °C.

The morphology and size of Fe_3_O_4_@SiO_2_-SH nanoparticles were probed by scanning electron microscopy by using a Jeol5600LV apparatus with a detector of secondary electrons. To increase its conductivity for analysis, the sample was coated with a thin gold layer with thickness of 0.2 nm by employing a Balzers sputterer.

Hydrodynamic size, PI, and zeta potential of Fe_3_O_4_@SiO_2_-SH nanoparticles in different media were determined by dynamic light scattering (DLS) on a Horiba NanoPartica SZ-100 Series instrument. Suspensions of the particles were prepared in both deionized water and complete cell culture medium at the same concentration of 0.4 mg/mL and were agitated by ultrasound for 25 min before the measurement. The suspensions were measured in disposable plastic cuvettes at temperature of 25 °C with a scattering angle of 173°. Each measurement was performed in at least 12 repeats after 30 s, and the experimental data were processed by the software supplied with the SZ-100 instrument.

### 4.3. Cell Cultures and Culture Conditions

A549 cells were cultured in Minimum Essential Medium Eagle (MEM) with L-glutamine and sodium bicarbonate (Sigma-Aldrich, St. Louis, MO, USA) in the presence of 10% fetal calf serum, 1 mM pyruvate, 10 mM HEPES, 50 μg/mL penicillin, and 50 μg/mL streptomycin (all supplements from Life Technologies, Grand Island, NY, USA). MCF-7 cells were maintained in MEM alpha modification with L-glutamine and sodium bicarbonate (Sigma-Aldrich, St. Louis, MO, USA) supplemented with 10% fetal calf serum, 1 μg/mL insulin, 50 μg/mL penicillin, and 50 μg/mL streptomycin (all reagents from Life Technologies, Grand Island, NY, USA). HepG2 were propagated in Dulbecco’s Modified Eagle’s Medium DMEM—a high glucose medium (Sigma-Aldrich, St. Louis, MO, USA)—supplemented with 10% (v/v) fetal bovine serum, 2 mM L-glutamine, 1 mM pyruvate, 10 mM HEPES, 10 µL/mL MEM non-essential amino acids, 50 µg/mL penicillin, and 50 µg/mL streptomycin (all reagents from Life Technologies, Grand Island, NY, USA). A549, MCF-7, and HepG2 cells were purchased from the European Collection of Cell Cultures (ECACC, Salisbury, UK). The cell cultures were maintained under standard cell culture conditions at 37 °C in a humidified incubator under an atmosphere of 5% CO_2_ and 95% air. Cells were passaged every 2–3 days to obtain exponential growth. The cells were maintained in the culture for no more than 20 passages.

### 4.4. Real-Time Cell Proliferation and Adhesion Assays with the xCELLigence System

The RTCA SP xCELLigence system (Roche and ACEA Biosciences, San Diego, CA, USA) was used to monitor cell adhesion, proliferation, and cytotoxicity on A549, HepG2, and MCF-7 cells treated with Fe_3_O_4_@SiO_2_-SH nanoparticles. The system had been tested by a Resistor Plate before the RTCA Single Plate station was placed inside the incubator at 37 °C with 5% CO_2_. First, the optimal seeding concentration for experiments with the A549, HepG2, and MCF-7 cells was determined. Background measurements were taken by adding 100 µL of an appropriate medium to the wells of an E-Plate 96. Cell suspension (90 µL) was added to each well of the E-plate 96 at the cell density of 4000 (A549), 30,000 (HepG2), and 8000 (MCF-7) cells per well. The cells were monitored every 30 min by the xCELLigence system. Approximately 24 h later, when the cells were in the log growth phase, the cells were exposed in triplicates to 10 µL of sterile deionized water with Fe_3_O_4_@SiO_2_-SH nanoparticles to obtain the final desired concentration in each well. Negative controls received sterile deionized water for cell cultures (Lonza, Basel, Switzerland), whereas cells treated with 5% DMSO were used as positive controls. The dynamic cell adhesion, proliferation, and cytotoxicity of the particles on A549, HepG2, and MCF-7 cells was monitored for at least 72 h. Data analysis was performed by using xCELLigence 1.2.1 software (Roche and ACEA Biosciences, San Diego, CA, USA).

### 4.5. Trypan Blue Exclusion Test for Cell Proliferation and Viability

A549 cells were seeded at a concentration 5 x 104 cells/1 mL and treated with 1, 10, 50, 100, and 200 µg/mL of Fe_3_O_4_@SiO_2_-SH nanoparticles. Cells treated with doxorubicin, the topoisomerase II inhibitor, at concentrations of 0.25 and 0.5 µM were used as a positive control. Cell proliferation and viability were determined 48 h following the treatment. The cells were detached by incubation with 0.05% trypsin-EDTA (Life Technologies, Grand Island, NY, USA) for 8 min. The trypsin-detached cells were pooled with the medium containing floating cells. Cell membrane integrity was determined based on the Trypan blue exclusion technique by mixing 50 μL of 0.4% Trypan blue (Sigma-Aldrich, St. Louis, MO, USA) and 50 μL of a cell suspension. Cell counts were carried out by using a Bürker chamber and light microscope Nikon Eclipse E200 (Nikon, Tokyo, Japan).

### 4.6. Cell Cycle Distribution and Internucleosomal DNA Fragmentation Analysis

Where the cell cycle distribution analysis is concerned, the cells were washed with ice-cold PBS and fixed with 70% (v/v) ethanol. In order to detect low-molecular-weight fragments of DNA, the cells were incubated for 5 min at room temperature in a buffer (192 mL of 0.2 M Na_2_HPO_4_ + 8 mL of 0.1 M citric acid, pH 7.8) and then labelled with propidium iodide in Vindelov’s solution for 1 h at 37 °C. DNA content was determined by using the flow cytometer CytoFLEX LX Flow Cytometer (Beckman Coulter, Miami, FL, USA) with an excitation wavelength of 488 nm. The list mode data were analyzed by the Kaluza Analysis 2.1 software (Beckman Coulter, Miami, FL, USA).

### 4.7. Immunofluorescence Staining of β-Tubulin, Paxillin, and Actin

For each condition, 100,000 cells were seeded in 2-well chamber slides SPL (SPL Life Sciences, Pocheon-si, Korea). After seeding (usually 24 h later), the original medium was replaced with fresh medium, and the cells were treated with 50 and 200 μg/mL of Fe_3_O_4_@SiO_2_-SH nanoparticles. Cells treated with 0.5 μg/mL of cytochalasin D, a potent inhibitor of actin polymerization, were used as positive control. Following 24-h treatment, the cells were fixed with 4% freshly prepared paraformaldehyde for 10 min at room temperature, washed with PBS, permeabilized in 0.2% Triton X-100/PBS for 15 min at room temperature and washed with PBS (all reagents from Sigma–Aldrich, St. Louis, MO, USA). Before incubation with the primary antibody (overnight at 4 °C), the cells were incubated with 7% heat-inactivated fetal calf serum + 2% bovine serum albumin in PBS for 30 min at room temperature. Mouse monoclonal anti-β-tubulin antibody (Life Technologies, Grand Island, NY, USA) was used for β-tubulin detection or mouse monoclonal primary antibody anti-paxillin (Life Technologies, Grand Island, NY, USA) was applied for detection of paxillin. For the secondary antibody, the affinity pure donkey anti-mouse-tetramethylrhodamine (TRITC) conjugated antibody was purchased from the Jackson ImmunoResearch Laboratories (West Grove, PA, USA). The secondary antibody was applied to each slide (after their pre-incubation with 5.5% donkey serum in PBS for 30 min at room temperature) and incubated for 1 h in the dark. Cells were then washed three times with PBS and incubated with phalloidin Alexa Fluor 488 (Life Technologies, Grand Island, NY, USA) in 1% bovine serum albumin (BSA)/PBS (1:40 ratio of phalloidin Alexa Fluor 488 stock solution and 1% BSA/PBS solution) for 45 min at room temperature. The nuclei were counterstained with 100 μL of 4′,6-diamidino-2-phenylindole (DAPI) at 1 μg/mL for 20 min. After three washing cycles with PBS, the slides were mounted with an antifading ProLong^®^ Gold mounting medium (Life Technologies, Grand Island, NY, USA). The slides were examined by a Nikon epifluorescence microscope system Eclipse 80i; the exposure time and dynamic range of the camera in all channels were adjusted to the same values for all of the slides to portray quantitatively comparable images. Images were further processed, merged, and analyzed using NIS-Elements Advanced Research 5.11 (instrument and software from Nikon, Tokyo, Japan). For analysis of FA areas, images with paxillin staining were background-corrected and thresholded, FAs clusters were identified, and the total respective areas per cell were calculated.

### 4.8. Analysis of Apoptosis

Apoptosis was determined by a CellEvent™ Caspase-3/7 green ReadyProbes™ reagent (Life Technologies, Grand Island, NY, USA). Briefly, A549 cells were seeded at a density of 100,000 cells/chamber in 2-well glass chamber slides (SPL Life Sciences, Pocheon-si, Korea) and were treated with 50 μg/mL and 200 μg/mL Fe_3_O_4_@SiO_2_-SH nanoparticles for 24 h. Cells treated with 10 μM cisplatin were used as a positive control and cells treated with H_2_O were used as a negative control. After treatment, 2 drops/mL of the CellEvent™ Caspase-3/7 green ReadyProbes™ reagent were added to the treated cells and the slides were incubated for 30 min in a humidified incubator at 37 °C and 5% CO_2_. Apoptotic cells with activated caspase-3/7 show bright green nuclei, while cells without activated caspase 3/7 exhibit minimal fluorescence signal. These cells were observed using a Nikon epifluorescence microscope system Eclipse 80i; the exposure time and dynamic range of the camera in all channels were adjusted to the same values for all of the slides to portray quantitatively comparable images. Images were further analyzed using NIS-Elements Advanced Research 5.11 (instruments and software from Nikon, Tokyo, Japan).

### 4.9. Western Blot Analysis

Whole-cell lysates (Cell Lysis Buffer, Cell Signaling Technology, Danvers, MA, USA) were prepared 48 h following the treatment of A549 cells with 50 and 200 µg/mL of Fe_3_O_4_@SiO_2_-SH nanoparticles. Cells treated with 5% PBS were used as a negative control. Cells treated with 0.25 µM doxorubicin were used as a positive control. Quantification of the protein content was performed by using the BCA assay (Sigma-Aldrich, St. Louis, MO, USA). The lysates (20 µg of purified protein) were loaded into lanes of polyacrylamide gel. After electrophoresis separation, the proteins were transferred to a polyvinylidene fluoride (PVDF) membrane (Bio-Rad, Hercules, CA, USA). Any non-specific bindings of the membranes were blocked for 1 h in a Tris-buffered saline (TBS) containing 0.05% Tween 20 and 10%, w/v, non-fat dry milk. The membranes were washed in TBS. Incubation with a primary antibody against specific antigens (Chk1, Chk1_serine 345—Cell Signalling, Danvers, MA, USA; Chk2, Chk1_threonine 68—Cell Signalling, Danvers, MA, USA; Rb, Rb_serine 807, and serine 811—Cell Signalling, Danvers, MA, USA; FAK, pFAK_ tyrosine 397—Cell Signalling, Danvers, MA, USA; β-actin—Sigma-Aldrich, St. Louis, MO, USA; ERK1/2, pERK1/2_threonine 202, and tyrosine 204—Cell Signalling, Danvers, MA, USA; p38, p38_ threonine 180, and tyrosine 182—Cell Signalling, Danvers, MA, USA; PCNA—Sigma-Aldrich, St. Louis, MO, USA) was performed at 4 °C overnight. The following day the membranes were washed 5 times with TBS, each time for 5 min, and once with TBS for 10 min, and then incubated with an appropriate secondary antibody (DakoCytomation, Glostrup, Denmark) for 1 h at room temperature. Band detection was performed by means of a chemiluminescence detection kit (Roche, Basel, Switzerland). To ensure equal protein loading, each membrane was reprobed and β-actin was detected.

### 4.10. Statistical Analysis

The descriptive statistics of the results were calculated and the charts were made using either Microsoft Office Excel 365 (Microsoft, Redmond, WA, USA) or GraphPad Prism 7 biostatistics (GraphPad Software, San Diego, CA, USA) software. In this study, all the values were expressed as arithmetic means with the SD of triplicates, unless otherwise noted. For quantitative data, normality testing was performed to assess whether parametric or nonparametric tests should be used. For experiments with parametric variables, the significant differences between the groups were analyzed using the Student’s t-test and a *p*-value < 0.05 was considered significant.

## Abbreviations

MNPs	Magnetic nanoparticles
MPI	Magnetic particle imaging
MSA	Mercaptosuccinic acid
DMSA	Dimercaptosuccinic acid
FT-IR	Fourier transform infrared spectroscopy
DSC	Differential scanning calorimetry
SEM	Scanning electron microscopy
DMSO	Dimethyl sulfoxide
DAPI	4’,6-diamidino-2-phenylindole
ECM	Extracellular matrix
DLS	Dynamic light scattering
PI	Polydispersity index
FAK	Focal adhesion kinase
FAs	Focal adhesions
Ph	Optical phase-contrast microscopy
MAPK	Mitogen-activated protein kinases
ERK	Extracellular signal regulated kinase
p38	p38 MAP kinase
ATR/Chk1	Ataxia telangiectasia and Rad3-related protein kinase/checkpoint kinase 1
ATM/Chk2	Ataxia-telangiectasia mutated kinase/checkpoint kinase 2
Rb	Retinoblastoma protein
PBS	Phosphate-buffered saline
TBS	Tris-buffered salineMinimum Essential Medium Eagle
MEM	Dulbecco’s Modified Eagle’s Medium
DMEMTRITC	Tetramethylrhodamine
BSA	Bovie serum albumin
PVDF	Polyvinylidene fluoride
